# Atomic layer deposition of high-density Pt nanodots on Al_2_O_3_ film using (MeCp)Pt(Me)_3_ and O_2_ precursors for nonvolatile memory applications

**DOI:** 10.1186/1556-276X-8-80

**Published:** 2013-02-15

**Authors:** Shi-Jin Ding, Hong-Bing Chen, Xing-Mei Cui, Sun Chen, Qing-Qing Sun, Peng Zhou, Hong-Liang Lu, David Wei Zhang, Chen Shen

**Affiliations:** 1 State Key Laboratory of ASIC and System, School of Microelectronics, Fudan University, Shanghai, 200433, China

**Keywords:** Atomic layer deposition, Pt nanodots, Nonvolatile memory

## Abstract

Pt nanodots have been grown on Al_2_O_3_ film via atomic layer deposition (ALD) using (MeCp)Pt(Me)_3_ and O_2_ precursors. Influence of the substrate temperature, pulse time of (MeCp)Pt(Me)_3_, and deposition cycles on ALD Pt has been studied comprehensively by scanning electron microscopy, transmission electron microscopy, and X-ray photoelectron spectroscopy. Therefore, Pt nanodots with a high density of approximately 2 × 10^12^ cm^-2^ have been achieved under optimized conditions: 300°C substrate temperature, 1 s pulse time of (MeCp)Pt(Me)_3_, and 70 deposition cycles. Further, metal-oxide-semiconductor capacitors with Pt nanodots embedded in ALD Al_2_O_3_ dielectric have been fabricated and characterized electrically, indicating noticeable electron trapping capacity, efficient programmable and erasable characteristics, and good charge retention.

## Background

Platinum (Pt) nanodots or nanoparticles have been attracting more and more attention due to their various potential applications. As a catalyst, Pt nanodots have been extensively used in the petroleum reforming and petrochemical industries as well as in fuel cells because of their excellent catalytic activity [1-4]. On the other hand, Pt nanodots have also been investigated for memory devices that utilize discrete metal nanodots as charge storage medium [5,6]. This is attributed to the potential that the nanodot-based memories can lessen the impact of localized oxide defects, lateral coupling of charge storage layers between adjacent devices, and stress-induced leakage current [7]. Moreover, Pt has a high work function of 5.1 eV, low diffusivity, and excellent thermal stability [6-8]. Therefore, the employment of Pt nanodots can obtain a deep potential well in memory devices to ensure good data retention, together with good compatibility with CMOS processing. However, most researchers used high-temperature rapid thermal annealing (RTA) of ultrathin Pt films to achieve high-density Pt nanodots [5,8,9], which might cause the formation of an additional interfacial layer between the high-permittivity (high-*k*) tunnel layer and silicon substrate as well as crystallization of the tunnel layer.

In recent years, atomic layer deposition (ALD) of Pt nanoparticles have been investigated on various surfaces such as micron-sized porous silica gel particles [10], SrTiO_3_ nanocubes [11], WC [12], and SiO_2_ film [7]. However, most of them are used for catalyst. Although Novak et al*.* reported ALD Pt nanoparticles for memory applications, their study relates only to deposition cycles rather than the effect of substrate temperature and pulse time of the precursor on the growth behavior of Pt nanoparticles [7]. Moreover, the ALD technique is also attempted to grow other metallic nanodots for memory applications, such as Ru, WN, and RuO_*x*_ nanodots [13-15]. It is worthwhile to mention that by means of the ALD technique, high-density metal nanodots can be obtained at much lower temperatures compared to high-temperature RTA of ultrathin metal films [16,17]. On the other hand, to further improve retention time and ensure low-voltage operation, recent efforts have been focused on high-*k* dielectrics to replace SiO_2_ as a gate oxide in nanodot floating gate memories [6]. Among high-*k* dielectrics, Al_2_O_3_ has been widely studied due to its dielectric constant of approximately 9, a large bandgap of 8.9 eV, a large band offset of 2.8 eV with respect to silicon, good chemical and thermal stabilities with the silicon substrate, and amorphous matrix at high temperature [18]. Therefore, in this article, the ALD growth of Pt nanodots on Al_2_O_3_ films has been investigated comprehensively, and the experimental parameters are optimized for high-density Pt nanodots. Further, metal-oxide-semiconductor (MOS) capacitors with Pt nanodots have been fabricated, and the corresponding memory characteristics are measured.

## Methods

Firstly, around 8-nm Al_2_O_3_ films were deposited on cleaned P-type silicon substrates by ALD using the precursors Al(CH_3_)_3_ and water. Subsequently, the ALD growth of Pt nanodots were carried out on the surface of Al_2_O_3_ film using (MeCp)Pt(Me)_3_ and O_2_ precursors in a commercial tool (TFS 200, Beneq, Vantaa, Finland). Herein, the precursor (MeCp)Pt(Me)_3_ was kept at 70°C, the vapor of which was pulsed into the reaction chamber by the carrier gas argon (99.999%). High-purity O_2_ (99.999%) was pulsed into the reaction chamber through a separate gas line with a flow rate of 100 sccm. During the ALD process, the working pressure in the deposition chamber was maintained at 5 mbar, and the O_2_ pulse time was fixed at 0.1 s. To obtain the optimal process conditions, the influences of substrate temperature, pulse time of (MeCp)Pt(Me)_3_, and reaction cycles on Pt nanodot growth were investigated respectively. Further, to investigate the characteristics of Pt nanodots as charge storage nodes, the Al gate MOS capacitors with 8-nm Al_2_O_3_/Pt nanodots/24-nm Al_2_O_3_ were fabricated; herein, Pt nanodots were deposited under optimized conditions (shown later). As a comparison, a MOS capacitor without Pt nanodots was also fabricated.

The thicknesses of Al_2_O_3_ film was measured by an ellipsometer (SOPRA GES 5E, Courbevoie, France). ALD of Pt was characterized by field emission scanning electron microscope (FE-SEM; JSM-6700 F, JEOL, Tokyo, Japan), high-resolution transmission electron microscope (HR-TEM), and X-ray photoelectron spectroscopy (XPS) (Kratos Axis Ultra DLD). Capacitance-voltage (*C*-*V*) measurements were performed on a LCR meter (Keithley 590, Cleveland, OH, USA), and voltage pulses were generated by a pulse/pattern generator (Keithley Model 3402).

## Results and discussion

### Impact of substrate temperature on ALD Pt nanodots

**Figure 1:**
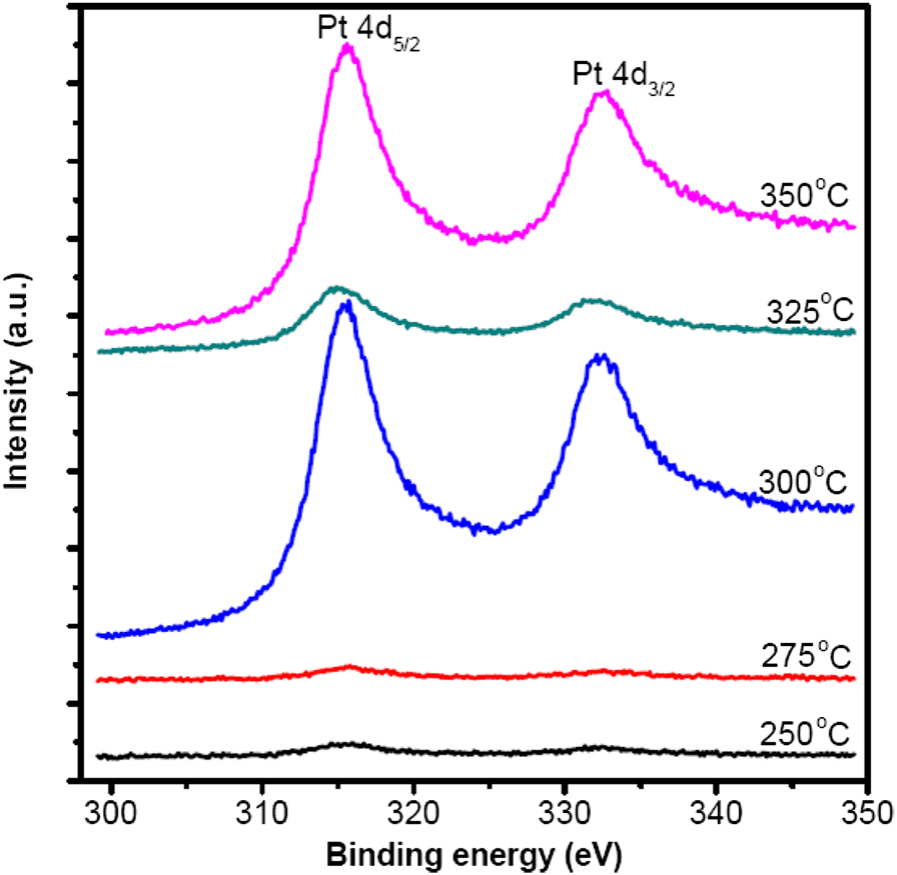
**Pt 4*d *XPS spectra of ALD Pt on Al**_**2**_**O**_**3**_**film at different substrate temperatures.** Deposition cycles 70.

Figure 1 shows the Pt 4*d* XPS spectra of the deposited Pt at different substrate temperatures. It is found that the peaks of Pt 4*d* are negligible in the case of 250°C and 275°C, indicating the growth of a few Pt atoms. Aaltonen et al*.* also reported that only very thin Pt films were obtained at 250°C compared to the deposition temperature of 300°C [19]. This could be attributed to the factor that low temperature cannot stimulate effectively the half reaction between (MeCp)Pt(Me)_3_ and Pt-O_*x*_, which is described as CH_3_C_5_H_4_Pt(CH_3_)_3_ + Pt-O_*x*_ → Pt (s) + CO_2_ (g) + H_2_O (g) + other by-products, where the Pt-O_*x*_ species represents oxygen adsorbed on the Pt surface [20]. When the substrate temperature was increased to 300°C, very strong photoelectron peaks associated with Pt 4*d*_5/2_ and 4*d*_3/2_ were observed, indicating the deposition of a mass of Pt atoms. However, the Pt 4*d* peaks decreased again when the substrate temperature was increased to 325°C, revealing a reduced deposition of Pt. As suggested in [19], during the following (MeCp)Pt(Me)_3_ pulse, the ligands react with the adsorbed oxygen layer. However, it is reported that oxygen can be desorbed from a Pt surface at 330°C [21]; therefore, it is likely that oxygen desorption also occurs at 325°C in our case. This will lead to a limited amount of oxygen on the Pt surface, thus reducing the reaction probability and the deposition of Pt as well. On the other hand, the thermal decomposition of (MeCp)Pt(Me)_3_ can also take place to some extent at a substrate temperature of 325°C [19]; this results in an additional deposition of Pt. In a word, the behavior of ALD Pt was determined by the aforementioned two competitive processes, and the former is likely dominant in the present experiment. When the substrate temperature goes up to 350°C, the resulting Pt 4*d* peaks become strong again. This should be ascribed to thermal decomposition of (MeCp)Pt(Me)_3_, thus resulting in the deposition of a mass of Pt atoms, as reported in the literature [19,22,23].

**Figure 2:**
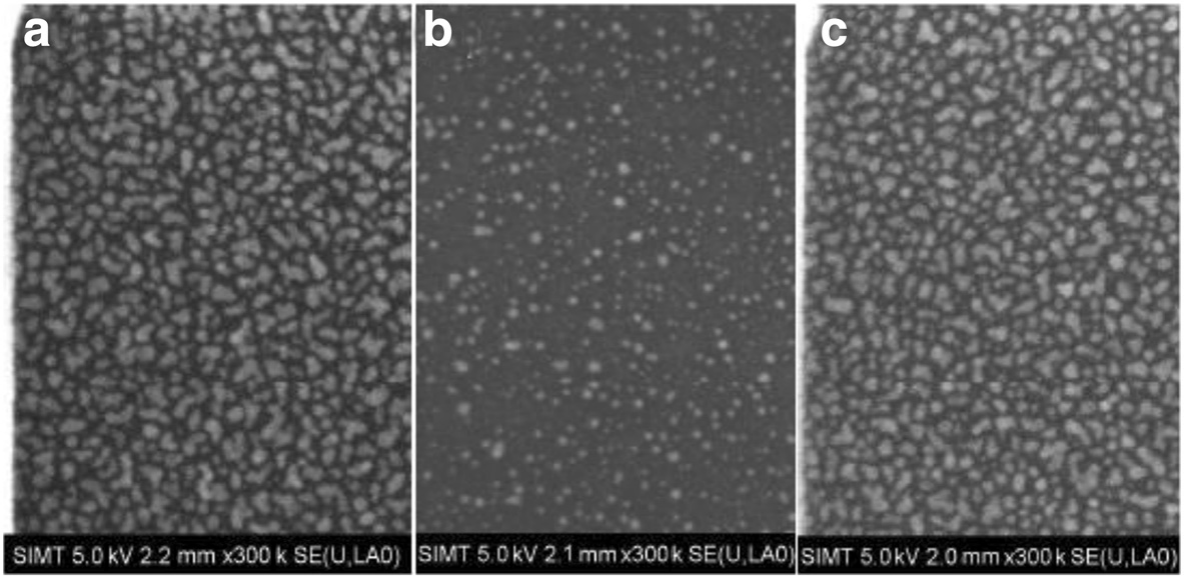
**SEM images of ALD Pt on the Al**_**2**_**O**_**3 **_**surface corresponding to different substrate temperatures.** (**a**) 300°C, (**b**) 325°C, and (**c**) 350°C.

In order to observe intuitively the formation of Pt nanodots, the surface morphologies of the Pt samples deposited at different temperatures were measured by SEM. In terms of substrate temperatures of 250°C and 275°C, the resulting SEM images do not show any nanodots (not shown here). Regarding the substrate temperature of 300°C, lots of Pt nanodots are observed on the surface of Al_2_O_3_, as shown in Figure 2a. When the substrate temperature increased to 325°C, the density and size of the deposited Pt nanodots became small, see Figure 2b. As the substrate temperature rose to 350°C, the resulting Pt nanodots become denser and bigger again, shown in Figure 2c. The aforementioned phenomena are in good agreement with the XPS spectra in Figure 1. Consequently, to achieve high-density Pt nanodots, the substrate temperature of 300°C is much preferred.

### Influence of (MeCp)Pt(Me)_3_ pulse time on ALD Pt nanodots

**Figure 3:**
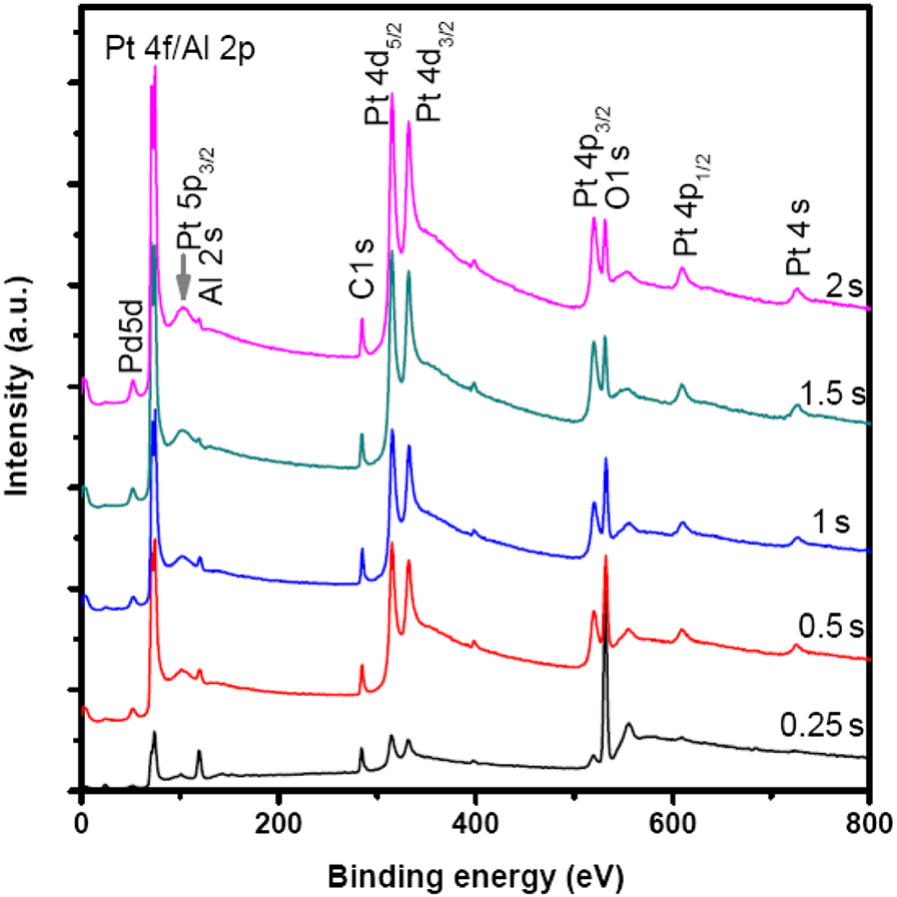
**Survey XPS spectra of ALD Pt on Al**_**2**_**O**_**3 **_**film as a function of (MeCp)Pt(Me)**_**3**_**pulse time.** Substrate temperature 300°C, deposition cycles 70.

With respect to a real ALD process, it is very important to employ enough pulse lengths of precursors to saturate the surface adsorption and ensure the monolayer growth. However, as for the growth of high-density metal nanodots, the density of Pt nuclei on the substrate surface is a key point, which depends on the substrate surface chemistry, the precursor activities, and the pulse length. In general, when the Pt nuclei at the surface are very dense, the resulting Pt might be in the form of a film. Contrarily, if the Pt nuclei are very sparse, the deposited Pt appears in the form of nanodots with a low density, which will not be able to meet the requirement of a memory device. Therefore, to achieve high-density Pt nanodots, the influence of the pulse time of (MeCp)Pt(Me)_3_ on ALD Pt nanodots should be investigated while maintaining constant O_2_ pulse time. Figure 3 shows the survey XPS spectra of the deposited Pt samples corresponding to different pulse times of (MeCp)Pt(Me)_3_ in the case of 70 deposition cycles. It is seen that the intensity ratio of Pt 4*p*_3/2_ to O 1*s* peaks increases distinctly with an increase of the (MeCp)Pt(Me)_3_ pulse time from 0.25 s to 1.5 s. This reflects a marked increase of Pt coverage on the surface of the Al_2_O_3_ film. When the pulse time is further increased to 2 s, the aforementioned intensity ratio exhibits a slight increase. Meanwhile, it is observed that the peaks of Pt 4*d* exhibit remarkable enhancement in comparison with those corresponding to 1.5-s pulse time. This indicates that when the pulse time exceeds 1.5 s, the Pt deposition is dominated by its growth on the surface of Pt nanodots due to the fact that most of the Al_2_O_3_ surface has been covered by ALD Pt, thus likely leading to the preferential vertical growth of Pt.

**Figure 4:**
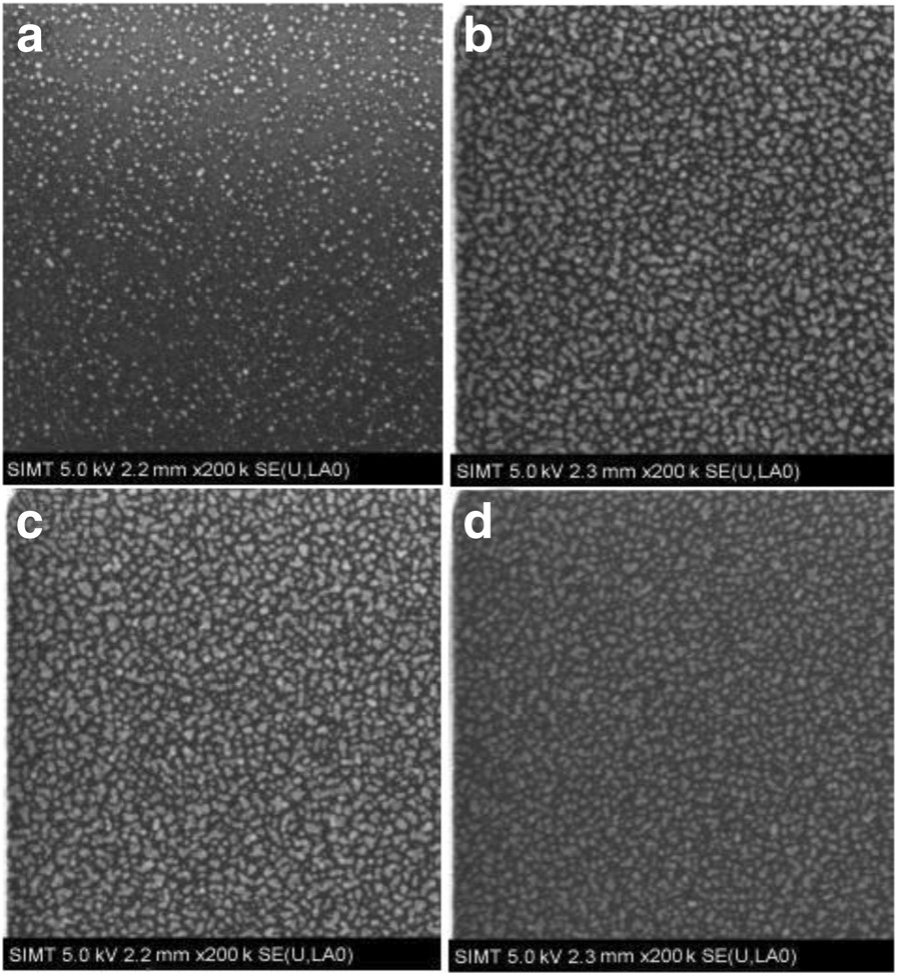
**SEM images of ALD Pt on Al**_**2**_**O**_**3 **_**for different pulse times of (MeCp)Pt(Me)**_**3**_**.** (**a**) 0.25, (**b**) 0.5, (**c**) 1, and (**d**) 2 s (substrate temperature 300°C, deposition cycles 70).

Figure 4 shows the surface SEM images of the deposited Pt nanodots corresponding to different pulse times of (MeCp)Pt(Me)_3_ respectively. In the case of 0.25-s pulse time, the resulting Pt nanodots are very small, sparse, and nonuniform. Nevertheless, when the pulse time increases to 0.5 s, the resulting Pt nanodots become much denser and bigger, thus revealing that the pulse time of (MeCp)Pt(Me)_3_ plays a key role in the growth of Pt nanodots. Further, as the pulse time increases gradually to 2 s, the resulting Pt nanodots do not exhibit distinct changes based on the SEM images, but it is believed that the distances between nanodots become narrower and narrower, and even the coalescence between adjacent nanodots could occur. Therefore, to ensure the growth of high-density Pt nanodots, the coalescence between adjacent nanodots should be avoided during ALD. For this purpose, the pulse time of (MeCp)Pt(Me)_3_ should be controlled between 0.5 and 1 s.

### Influence of deposition cycles on ALD Pt

**Figure 5:**
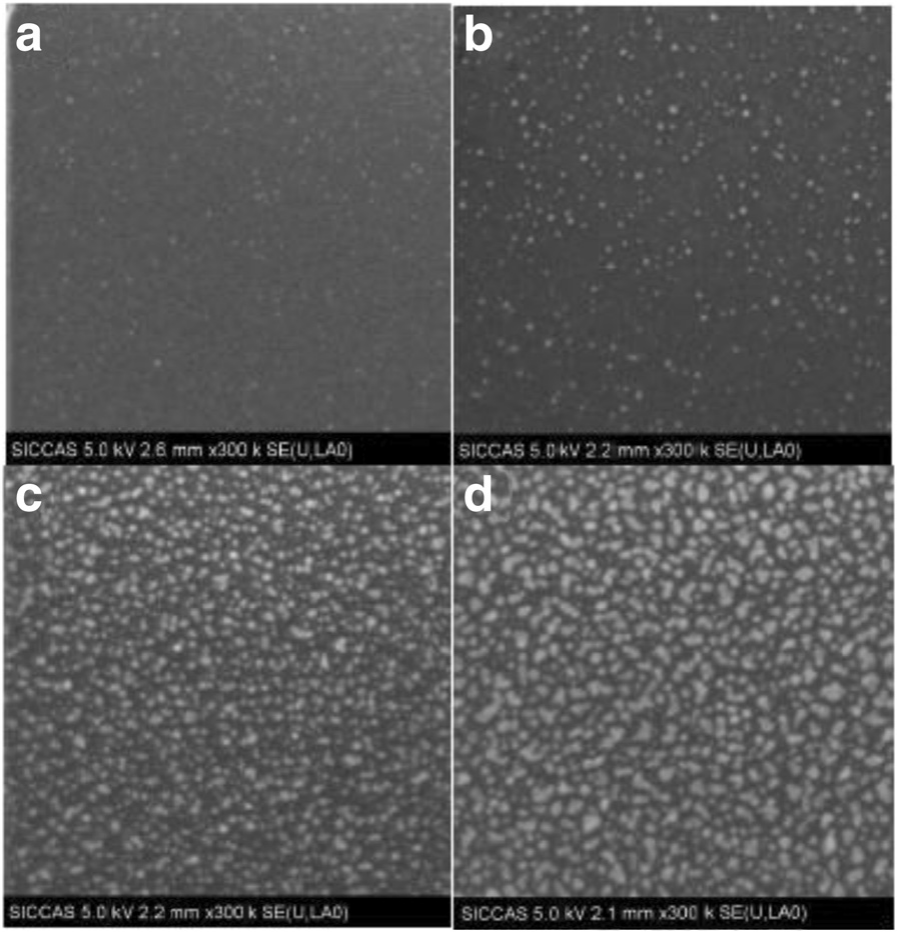
**SEM images of ALD Pt on Al**_**2**_**O**_**3 **_**as a function of deposition cycles.** (**a**) 40, (**b**) 60, (**c**) 70, and (**d**) 90 cycles. Substrate temperature, 300°C; pulse time of (MeCp)Pt(Me)_3_, 1 s.

Figure 5 illustrates the surface morphologies of the resulting Pt nanodots as a function of deposition cycles. In the case of ≤60 deposition cycles, the deposited Pt nanodots exhibit low densities and small dimensions. When the number of deposition cycles increases to 70, the density of Pt nanodots increases remarkably. As the deposition duration reaches 90 cycles, the resulting Pt nanodots exhibit much larger dimensions and irregular shapes as well as a reduced density.

**Figure 6:**
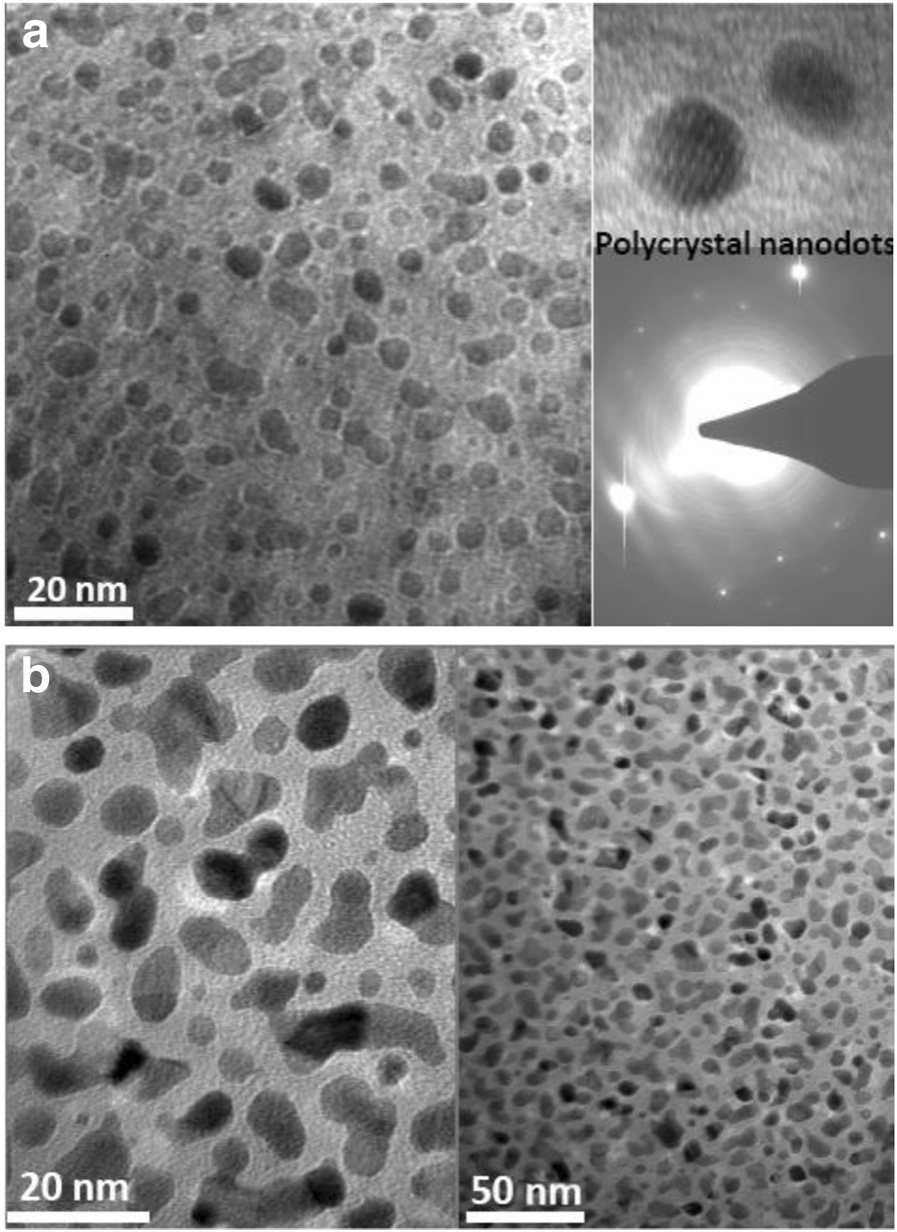
**Planar TEM images of ALD Pt on Al**_**2**_**O**_**3 **_**film.** Corresponding to (**a**) 70 cycles, together with an electron diffraction image of selected area, and (**b**) 90 cycles.

Further, the planar TEM images of the Pt nanodots corresponding to 70 and 90 cycles are compared in Figure 6. It is found that the Pt nanodots corresponding to 70 deposition cycles exhibit a density as high as approximately 2 × 10^12^ cm^-2^ and a well-separated distribution, and most of them appear in the form of a sphere. In addition, an electron diffraction image of the selected area shows that the Pt nanodots are polycrystalline. However, for 90 deposition cycles, the resulting Pt nanoparticles exhibit various irregular shapes such as sphere, ellipse, bar, etc. The observed decrease in the density of Pt nanoparticles should be attributed to the coalescence between adjacent nanodots, which is incurred by a long deposition time. Based on the above discussion, 70 deposition cycles are advisable to achieve high-density Pt nanodots on the surface of Al_2_O_3_. On the other hand, it should be noticed that the substrate surface has a great influence on the growth of metal nanodots. As an example, compared to the surface of ALD Al_2_O_3_ film, the surfaces of thermal SiO_2_ and H-Si-terminated silicon are not in favor of the growth of Pt and Ru nanodots and thus cannot achieve high-density nanodots [7,16]. This is due to the fact that the surface chemistry determines the initial nucleation of metal.

**Figure 7:**
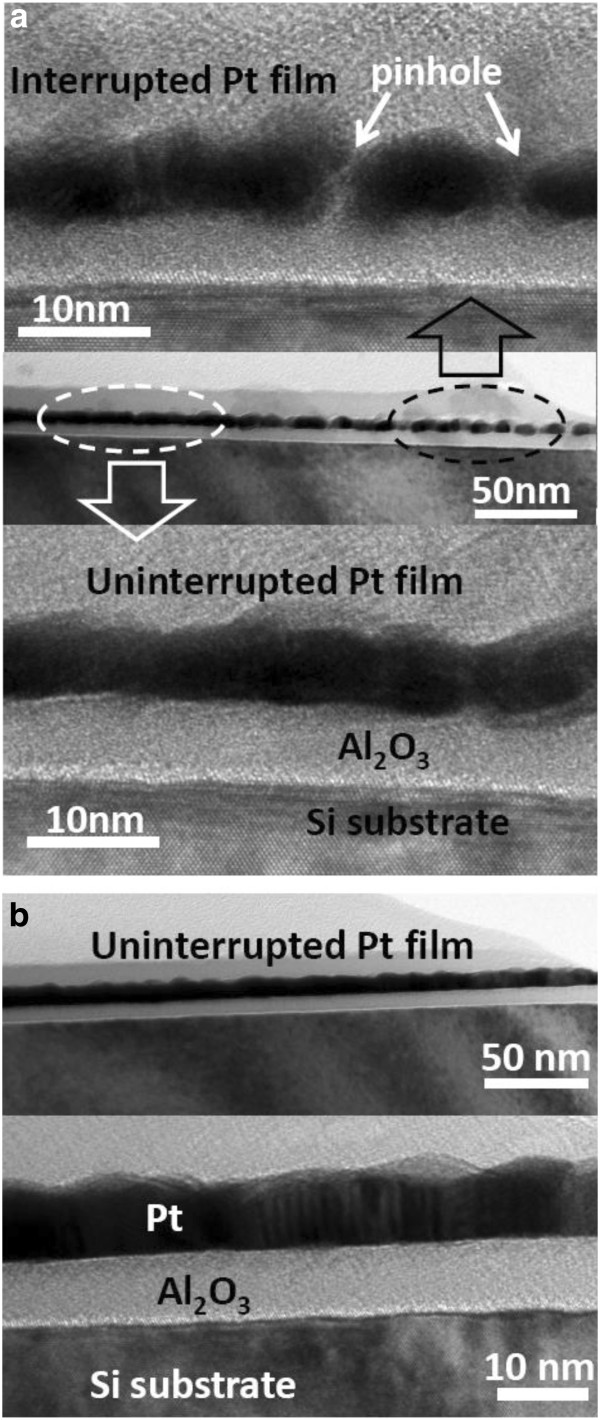
**Cross-sectional TEM images of ALD Pt corresponding to different deposition cycles.** (**a**) 120 and (**b**) 200 cycles.

As the deposition cycles increase continuously, the Pt particles become bigger and bigger, and the probability of coalescence between Pt particles increases gradually. As shown in Figure 7a, when the deposition cycles increase up to 120, a discontinuous Pt thin film is formed, i.e., the Pt film is interrupted by pinholes in some regions. Further, a perfect Pt film without any pinholes is formed when the deposition duration reaches 200 cycles, shown in Figure 7b.

### Memory characteristics of MOS capacitors with Pt nanodots

**Figure 8:**
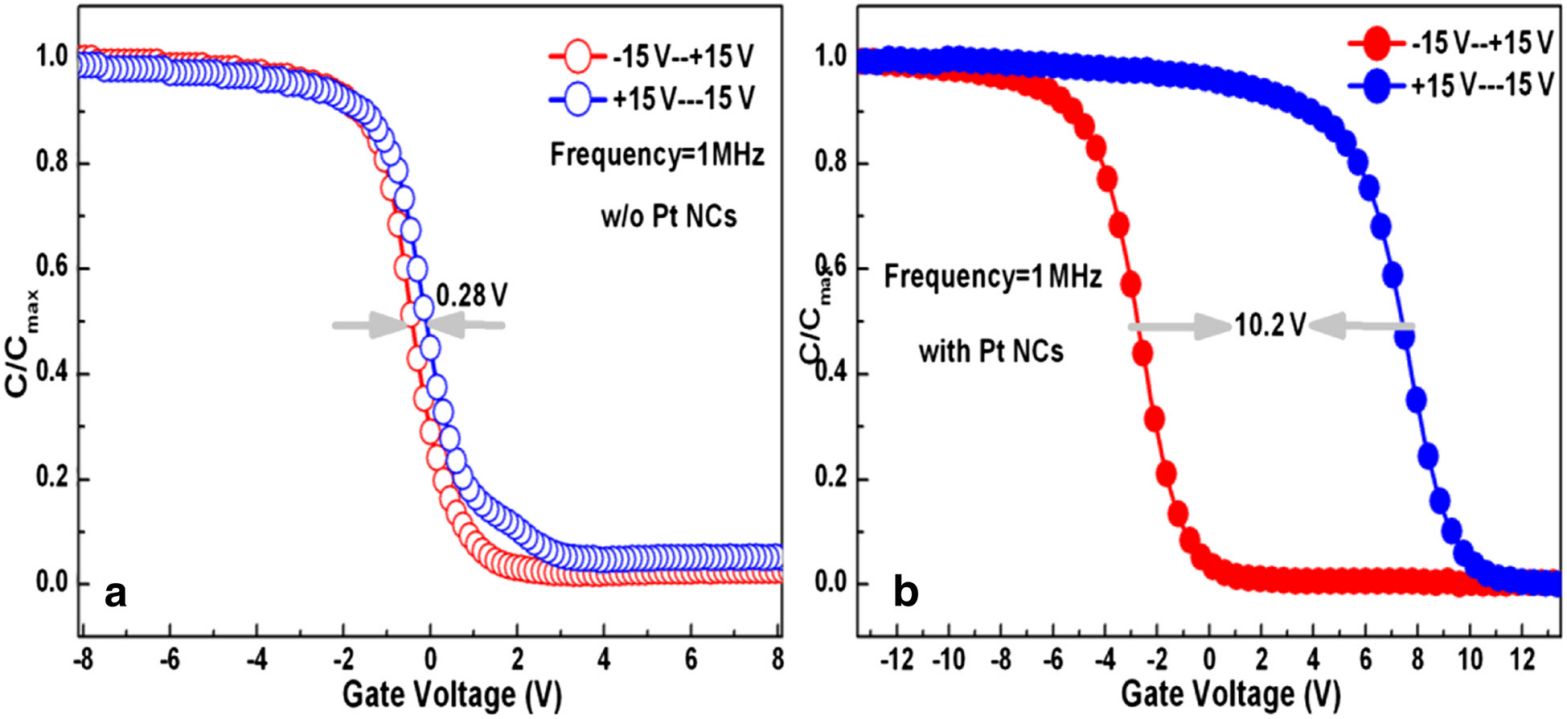
**High-frequency (1 MHz) *C*-*V *hysteresis curves of the MOS capacitors.** (**a**) Without Pt nanodots and (**b**) with Pt nanodots.

Figure 8 shows the *C*-*V* hysteresis curves of the MOS capacitor with Pt nanodots in comparison with the counterpart without Pt nanodots. It is indicated that the capacitor with Pt nanodots exhibits a hysteresis window as much as 10.2 V in the case of +15 V to -15 V of scanning voltage. However, the hysteresis window for the capacitor without Pt nanodots is as small as 0.28 V. This reveals that the Pt nanodots have significant charge trapping capability.

**Figure 9:**
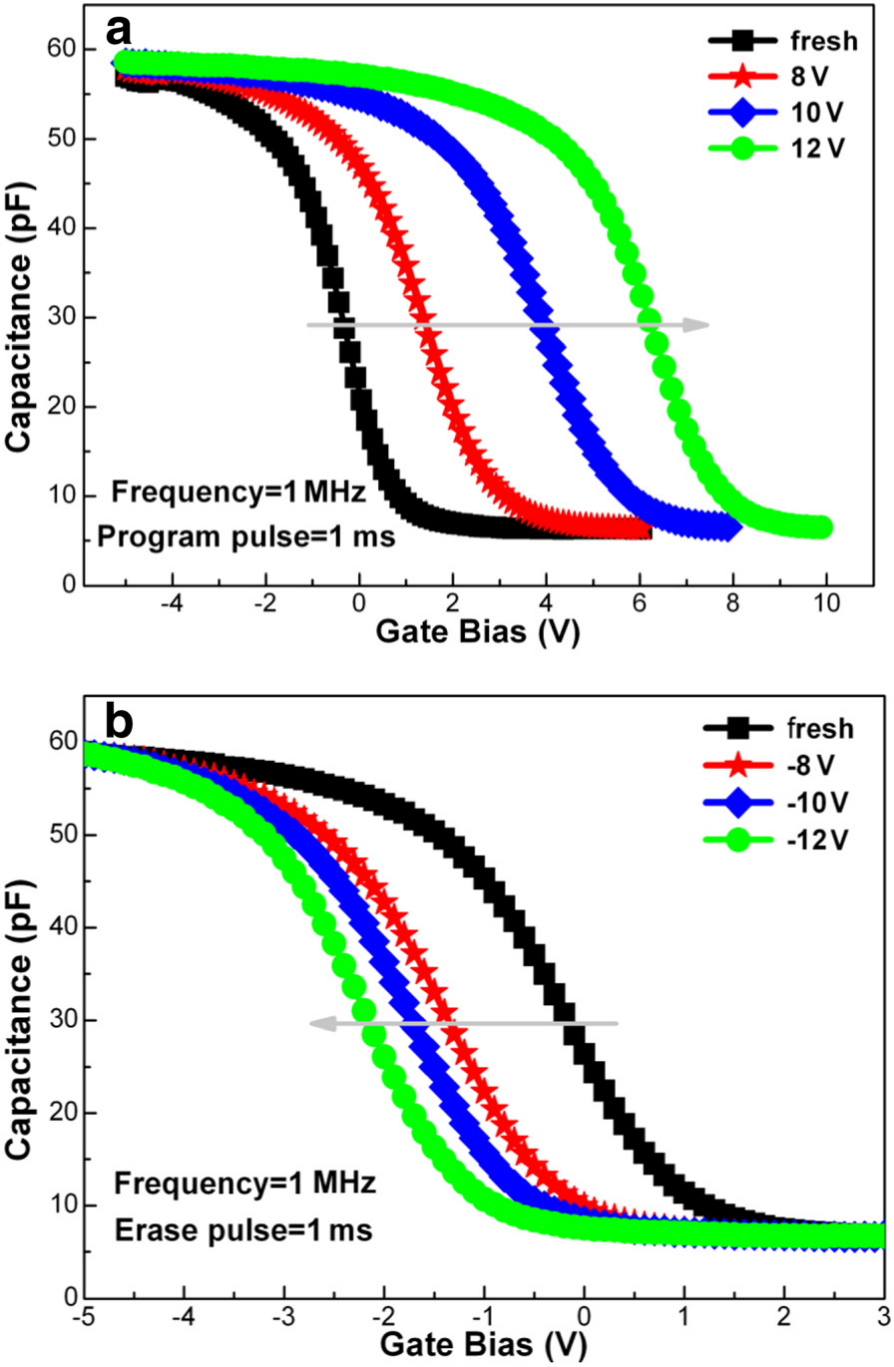
**High-frequency (1 MHz) *C*-*V *curves of the memory capacitor.** Corresponding to (**a**) programming and (**b**) erasing under different gate voltage for 1 ms, respectively.

In order to investigate the programmable and erasable characteristics of the memory capacitor, the MOS capacitor with Pt nanodots was programmed and erased, respectively, under different voltages for 1 ms, as shown in Figure 9. It is found that the resulting *C*-*V* curve shifts noticeably towards a positive bias with increasing the programming voltage from +8 to +12 V, see Figure 9a. This indicates that the probability of electrons injected from the substrate is enhanced with increasing the gate voltage, which is due to a reduction of the Fowler-Nordheim tunneling barrier through the tunneling Al_2_O_3_ layer. On the contrary, as the erasing voltage changes from -8 to -12 V, the resulting *C*-*V* curve moves gradually in the direction of negative bias, see Figure 9b. This reveals hole trapping and electron de-trapping in the MOS structure. In a word, our experimental results indicate that the MOS capacitor with Pt nanodots can be programmed and erased efficiently even under low voltages of ±8 V, and the resulting memory window is as large as 2.8 V for 1 ms of programming/erasing time.

**Figure 10:**
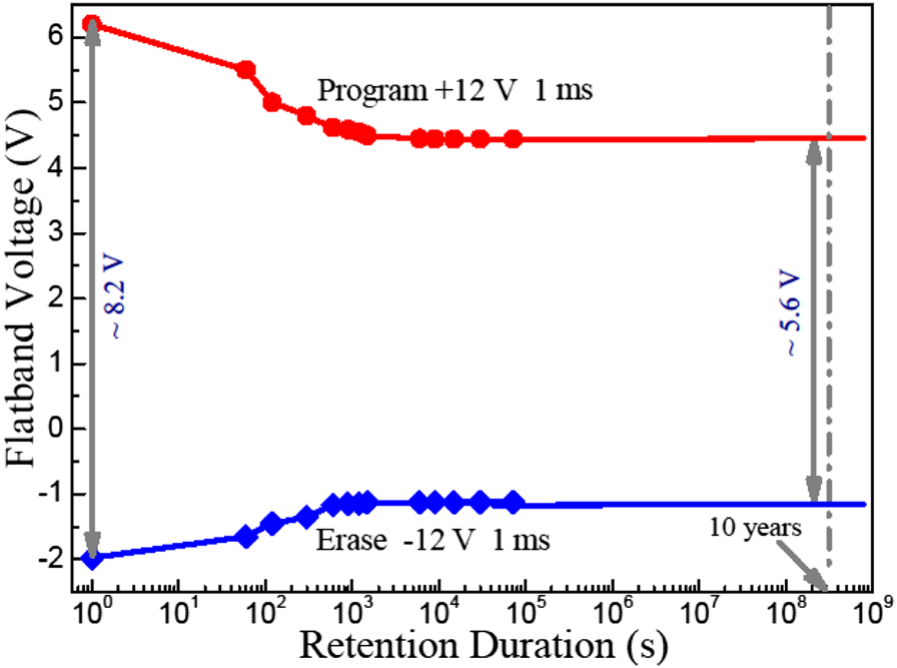
Charge retention characteristics of the MOS capacitor with Pt nanodots at room temperature.

Figure 10 shows the charge retention characteristics of the MOS capacitor with Pt nanodots at room temperature. It is seen that the memory window is close to 8.2 V after programming/erasing under ±12 V for 1 ms, and the deduced memory window still approaches 5.6 V after 10 years by extrapolation. This indicates that Pt nanodots can offer not only enough capability for electron storage but also good charge retention characteristic.

## Conclusions

Growth of Pt nanodots on the surface of Al_2_O_3_ has been investigated by ALD using (MeCp)Pt(Me)_3_ and O_2_ precursors. By optimizing substrate temperature, pulse time of (MeCp)Pt(Me)_3_, and deposition cycles, Pt nanodots with a high density of approximately 2 × 10^12^ cm^-2^ have been achieved, i.e., the process parameters are as follows: substrate temperature 300°C, (MeCp)Pt(Me)_3_ pulse time 1 s, and 70 deposition cycles. Further, the fabricated MOS capacitor with Pt nanodots exhibits noticeable programmable and erasable characteristics even under low voltages of ±8 V, a large memory window, and good charge retention at room temperature.

## Competing interests

The authors declare that they have no competing interests.

## Authors’ contributions

SJD carried out the main part of the experimental design and analytical works and drafted the manuscript. HBC carried out the fabrication and electrical measurements and some of the analytical works. XMC, SC, QQS, PZ, HLL, DWZ, and CS gave their good comments and suggestions during this study. All authors read and approved the final manuscript.
